# The high frequency of alcohol advertising during televised English Premier League football games shown in Ethiopia

**DOI:** 10.1186/s12954-022-00591-y

**Published:** 2022-02-05

**Authors:** Mulugeta Tamire, Alex Barker, Sefonias Getachew, Rachael L. Murray, Rihanna Amedala, John Britton, Wakgari Deressa, Andrew W. Fogarty

**Affiliations:** 1 Department of Preventive Medicine, School of Public Health, College of Health Sciences, Addis Ababa University, P.O. Box 7574, Addis Ababa, Ethiopia; 2 Department of Psychology, Nottingham Trent University, Nottingham, UK; 3 Division of Epidemiology and Public Health, University of Nottingham, Nottingham, UK

**Keywords:** Alcohol, Advertising, Ethiopia, Sport, Television

## Abstract

**Background:**

Excessive alcohol consumption is an important risk factor for increased morbidity, mortality and other social harms globally. Televised sport allows the promotion of alcoholic drinks to a large and often young audience, and thus can be used to develop new markets for alcohol in low- and middle-income countries. This study aimed to analyse the alcohol advertising displayed during televised English Premier League (EPL) games, which is widely viewed in Ethiopia, and are particularly popular among young people.

**Methods:**

Nineteen live televised EPL football matches broadcast in Ethiopia on the SuperSport channels over 4 weeks of the 2018/19 season were digitally recorded from the digital satellite television. Exposure to alcohol advertising was measured by calculating the total elapsed time duration with the alcohol content from the pre-match to the end of the post-game period of the broadcast.

**Results:**

Data were available for a total of 2451 min broadcast time. Alcohol advertising accounted for 205.2 min (8.4%) with a mean duration of 10.8 min per match (range 5.5 to 22.6). The dominant format of alcohol advertisement was the display of logos associated with an alcoholic drink on the television (TV) screen, which accounted for approximately 43% of the total alcohol advertising time. This was followed by pitch side virtual display (17.7%) and a glass of alcohol drink on the TV screen (17.6%). Over three quarters of alcoholic drink advertising (77.7%) was during active football playing time. None of the advertisements on the televised football games showed cigarettes.

**Conclusion:**

There is a high frequency of exposure to alcohol advertising during televised EPL matches in Ethiopia. It is important to ensure that the newly introduced domestic ban on alcohol advertising is also applied to foreign satellite broadcasters. This is likely to have relevance to other sub-Saharan African countries in promoting public health strategies to reduce harm from alcohol consumption.

## Background

Alcohol consumption caused over 3 million deaths globally in 2016, and over 5% of the global burden of disease and injury [1]. Alcohol content and advertising is regularly shown in televised sports programmes [2, 3]. SuperSport channels from the digital satellite television (DSTV), a sub-Saharan African direct broadcast satellite service owned by MultiChoice, which is the most watched football channel in Ethiopia [4], broadcasting all the matches of the league to most African countries including Ethiopia [5]. A systematic review of longitudinal studies reported that adolescent exposure to alcohol advertising predicts both the onset of drinking among non-drinkers and increased levels of consumption among existing drinkers [6]. Another large-scale national longitudinal study from the USA provided evidence of alcohol advertising expenditures and the degree of exposure to alcohol advertisements in the 15–26-year olds’ media environment was associated with higher levels of consumption of alcohol [7]. Furthermore, recent systematic reviews and descriptive synthesis of the available global literature showed positive associations between alcohol marketing and drinking among adolescents and young adults [8–10].

Ethiopia is a large East African country, with a Gross National Product of $783 per capita in 2016, and an economic growth rate of approximately 10% over the past decade [11]. The country has one of the fastest growing populations, with over 100 million individuals, where adolescents and youths aged 10–24 years, account for over one-third of the total population [12] representing a potentially valuable market for alcoholic drinks**.** The Federal Government of Ethiopia ratified the Food and Medicine Administration Proclamation (Proclamation No. 1112/2019) that bans all alcohol advertisements on local broadcast media and smoking in public places in February 2019 with a 3 months transition period to be fully implemented on 29 May 2019 [13]. This is consistent with the World Health Organization (WHO) global alcohol strategy that recommends reducing the impact of targeting alcohol marketing to areas which may appeal especially to young people [14] with a view to minimising unhealthy patterns of alcohol consumption and hence promoting a harm reduction public health strategy globally.

An association between alcohol companies sponsoring sports and alcohol consumption is well established [15]. We have recently reported that watching televised football is associated with drinking alcohol in adolescents living in Ethiopia [16]. Televised football is a popular leisure-time activity in Ethiopia and attracts a young audience [16], who often watch the football in public bars and/or sport houses. As sport, and in particular football, is often used as a vehicle for advertising, this is one way the alcohol industry can reach a large, young international audience and hence potential future customers. We aimed to measure the incidence of exposure to advertising of alcohol products via televised English Premier League (EPL) football games in Ethiopia at the time alcohol advertising had already been banned in the country.

## Methods

### Selection of broadcasts

The EPL was selected for this study as it is the most popular televised football league in Ethiopia [4], via SuperSport channels from the digital satellite television (DSTV). We purposively selected matches of the five most popular EPL teams in Ethiopia (Arsenal, Chelsea, Liverpool, Manchester City and Manchester United). The selection of the teams used the top five favourite football teams identified from responses of over 2500 high school students involved in our previous study from urban and rural parts of different regions in Ethiopia [16]. Accordingly, from the schedules of the leagues for the year 2018/19 we included all matches of the selected teams during weeks 34 to 37 or a 1-month duration from 13/04/2019 to 06/05/2019.

### Data collection

Televised football matches including active play, half-times intervals, pre- and post-match interviews, commentators’ discussions and advertisements shown before the start and after the end of the games (i.e. from the point the previous broadcast ends to when the next broadcast begins) were digitally recorded using two live recorders.

### Data coding and analysis

For each continual visual reference to alcohol, we coded the first time the image was visible and time it disappeared from screen. The lead author (MT) coded the recorded matches using a play and pause technique, where playing back to record the time the advertisement appeared/started and ended and find the elapsed time of the alcohol advertisement. After reading and recording of the exact start time and end time, the difference was taken as the duration of the advertisement. We coded all alcohol content that was clearly visible. When a direct view of the advertisement was interrupted or blocked by other displays, the whole content of the advertisement was counted as two separate appearances and considered as two different views. Alcohol types were categorised as beer, wine and whisky based on the appearances while the duration of all the alcohols together presented as total alcohol advertisement in the analysis. One of the co-authors (RA) re-coded two of the matches (with longest and shortest alcohol advertisement duration) to check for the reliability of the data. We also noted any cigarette industry advertising to ensure that the national ban [17], on this activity was being enforced.

We coded each match in a separate Microsoft Excel file along with general information about the match including match name, date, start time, time ended for each time categories considered in this analysis: Pre-match, first half, half-time break, second half and after game period. Data were then exported to IBM SPSS Statistics for Windows, Version 24.0 (IBM Corp., Armonk, N.Y., USA) for further analysis. We analysed the total and mean duration of alcohol advertisement during each match using descriptive statistics.

### Operational definitions

**On advertising wall:** large printed or drawn format in a static wall or fixed location within the stadium.

**Virtual pitch side:** Advertisements using digital technology and displaying virtual images that change in a given time on the sides of playfield.

**Glass/bottle on the TV screen:** A glass/bottle of alcoholic drink appearing on the TV screen that was superimposed by the satellite broadcaster to Ethiopia.

**Logo on TV Screen:** A logo of an alcohol drink appearing on the TV screen being superimposed by the satellite broadcaster to Ethiopia.

**Alcohol use on TV screen:** Visible appearance of people with alcoholic drinks.

**Verbal on TV screen:** Verbal advertisement about an alcohol drink presented on TV screen by the satellite broadcaster to Ethiopia.

**Text on TV screen:** Appearance of the name of an alcoholic drink on TV screen satellite broadcaster to Ethiopia.

## Results

### Advertisement duration by playtime of the matches

One of the matches (Manchester City versus Leicester City) was accidentally deleted from the recorder during data arrangement for the coding and hence 19 matches were available with data for analysis resulting in 2451 min of recorded TV for analysis (mean = 129 min per match). Of this, the overall time when alcohol advertisements were visible accounted for 205.2 min or 8.3% of the recorded time. Of the 205.2 min (mean 10.8 min per match), 159.8 (77.7%) occurred during active play and the remainder (46.0 [22.3%] minutes) outside active play. A slightly higher proportion of the alcohol advertisements were visible in the second half of matches (53.3%) compared with the first half (46.7%) (Table 1).

**Table 1:** Alcohol advertisements duration in minutes by play time of the matches

Match name	Pre-match	First half	Half-time	Second half	Post-match	In-play time	Out-of-play time	Total duration
Arsenal/Brighton	0.00	2.00	1.00	2.20	0.30	4.20	1.30	5.50
Arsenal/C. Palace	0.17	1.73	1.50	2.13	0.33	3.87	2.00	5.87
Burnley/Man City	0.75	9.12	1.75	10.65	0.33	19.77	2.83	22.60
C. Palace/Man City	0.18	4.85	1.83	4.42	0.73	9.27	2.75	12.02
Cardiff/Liverpool	0.57	1.97	2.00	2.15	0.27	4.12	2.83	6.95
Chelsea/Burnley	0.18	2.82	1.45	3.45	0.35	6.27	1.98	8.25
Chelsea/Watford	0.47	2.77	1.95	2.72	0.28	5.48	2.70	8.18
Everton/Man United	0.38	1.97	2.10	1.63	0.00	3.60	2.48	6.08
Leicester/Arsenal	0.20	8.37	2.03	10.15	0.00	18.52	2.23	20.75
Liverpool/Chelsea	0.43	3.67	2.52	5.07	0.27	8.73	3.22	11.95
Liverpool/Huddersfield	0.50	4.17	1.52	4.78	0.28	8.95	2.30	11.25
Man City/Spurs	0.25	2.28	1.47	2.55	0.00	4.83	1.72	6.55
Man United/Chelsea	0.28	7.85	2.05	9.35	0.28	17.20	2.62	19.82
Man United/Man City	0.15	4.18	2.07	5.22	0.25	9.40	2.47	11.87
Man United/West Ham	1.22	3.13	1.65	4.17	1.32	7.30	4.18	11.48
Watford/Arsenal	0.18	2.27	1.42	2.77	0.28	5.03	1.88	6.92
Wolves/Arsenal	0.00	2.30	.78	2.77	1.03	5.07	1.82	6.88
Newcastle/Liverpool	0.28	4.22	2.07	4.15	0.00	8.37	2.35	10.72
Huddersfield/Man United	0.00	5.07	2.07	4.78	0.23	9.85	2.30	12.15
Mean (minutes)	0.33	3.93	1.75	4.48	0.34	8.41	2.42	10.83
Total (%)	6.19 (3.0)	74.74 (36.3)	33.23 (16.2)	85.11 (41.4)	6.53 (3.2)	159.83 (77.7)	45.96 (22.3)	205.79

### Advertisements duration by the types of alcohol

As shown in Table 2, the overall duration of alcohol advertisement per football game ranged from 5.5 to 22.6 min with the mean duration of 10.8 min. Beer accounted for the majority (191.05 min (92.8%)) of the alcohol advertising time. We found no advertisements of cigarettes in any of the televised football matches. The duplicate coding process to check inter-reported reliability demonstrated 100% similarity between the coders by alcohol type, by location and in different play times.

**Table 2:** Advertisements duration by the types of alcohol

Match name	Beer	Wine	Whisky	All alcohol	Cigarette
Arsenal/Brighton	5.50	0.00	0.00	5.50	0.00
Arsenal/C. Palace	5.87	0.00	0.00	5.87	0.00
Burnley/Man city	22.60	0.00	0.00	22.60	0.00
C. Palace/Man City	12.02	0.00	0.00	12.02	0.00
Cardiff/Liverpool	6.95	0.00	0.00	6.95	0.00
Chelsea/Burnley	8.25	0.00	0.00	8.25	0.00
Chelsea/Watford	8.18	0.00	0.00	8.18	0.00
Everton/Man United	5.53	0.55	0.00	6.08	0.00
Leicester/Arsenal	20.75	0.00	0.00	20.75	0.00
Liverpool/Chelsea	11.95	0.00	0.00	11.95	0.00
Liverpool/Huddersfield	11.25	0.00	0.00	11.25	0.00
Man City/Spurs	6.55	0.00	0.00	6.55	0.00
Man United/Chelsea	15.68	1.42	2.72	19.82	0.00
Man United/Man City	7.18	1.45	3.23	11.87	0.00
Man United/West Ham	6.12	0.90	4.47	11.48	0.00
Watford/Arsenal	6.92	0.00	0.00	6.92	0.00
Wolves/Arsenal	6.88	0.00	0.00	6.88	0.00
Newcastle/Liverpool	10.72	0.00	0.00	10.72	0.00
Huddersfield/Man United	12.15	0.00	0.00	12.15	0.00
Mean (minutes)	10.06	0.23	0.55	10.83	0.00
Total (%)	191.05 (92.8)	4.32 (2.1)	10.42 (5.1)	205.79 (100)	0.00

### Alcohol advertisements by location

**Fig. 1:**
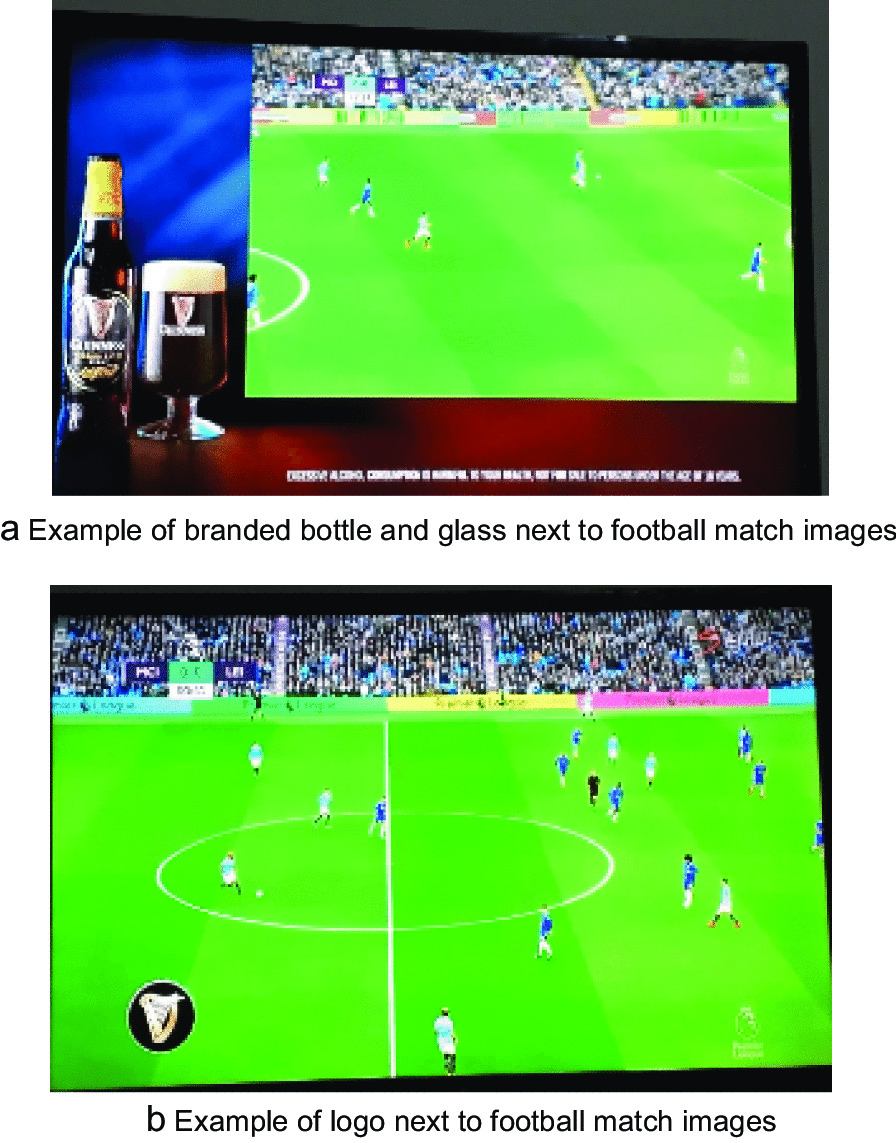
Examples of alcohol imagery observed in televised football matches in  Ethiopia 2019. Images from DSTV

The dominant type of alcohol advertisement was the display of the alcoholic drink logo on the TV screen (43% of all alcohol advertising time) followed by pitch side virtual display and pouring to the glass (17.7%) or displaying a full glass of alcohol on the TV screen (17.6%). Both the drink logo and the glass of alcohol displayed on the TV screen were superimposed on the original images by the satellite broadcaster (Fig. 1a, b). The advertisements added by the broadcaster also included inferred alcohol use (the visible appearance without consumption of an alcohol drink) by group of males and females sitting or standing in a bar, verbal or text advertisements out-of-playtime, consistently during the half-time break (Table 3).

**Table 3:** Alcohol advertisements by location

Match name	On sponsor wall	Virtual pitch side	Drink on TV screen	Logo on TV screen	People with drinks	Verbal on TV	Text on TV screen/Pitch side
Arsenal/Brighton	0.00	0.00	1.43	3.33	0.73	0.77	0.02
Arsenal/C. Palace	0.00	0.00	1.22	3.17	0.93	1.07	0.18
Burnley/Man City	0.00	6.83	2.22	12.95	0.97	1.00	0.00
C. Palace/Man City	0.00	5.18	1.80	3.27	1.43	1.80	0.72
Cardiff/Liverpool	0.00	0.00	2.10	3.40	0.97	1.13	0.73
Chelsea/Burnley	0.00	1.17	1.63	4.42	0.97	1.03	0.50
Chelsea/Watford	0.00	2.18	2.12	2.93	0.95	1.08	0.00
Everton/Man United	0.00	0.00	1.40	3.15	1.03	1.62	0.00
Leicester/Arsenal	0.00	4.93	1.55	12.77	0.97	1.48	0.00
Liverpool/Chelsea	0.00	0.97	2.55	3.02	0.97	1.48	0.00
Liverpool/Huddersfield	0.00	4.03	5.17	4.20	0.97	1.10	0.00
Man City/Spurs	0.00	0.00	1.63	3.95	0.97	.97	0.00
Man United/Chelsea	0.00	3.73	1.87	12.85	0.95	1.37	0.00
Man United/Man City	0.00	4.08	1.75	4.58	0.97	1.48	0.00
Man United/West Ham	0.00	3.27	3.67	3.33	0.93	.98	0.00
Watford/Arsenal	0.00	0.00	1.53	4.43	0.95	1.02	0.00
Wolves/Arsenal	0.00	0.00	1.68	4.25	0.95	.98	0.00
Newcastle/Liverpool	0.00	3.90	3.17	4.23	0.95	.98	0.00
Huddersfield/Man United	6.32	0.00	1.58	3.28	0.97	1.00	0.00
Mean (minutes)	0.33	2.12	2.11	5.13	0.98	1.18	0.11
Total (%)	6.32 (2.8)	40.27 (17.7)	40.07 (17.6)	97.51 (42.9)	18.53 (8.2)	22.34 (9.8)	2.15 (1.0)

## Discussion

This study presents the first data on the incidence of alcohol advertising that accompanies popular EPL games broadcast in Ethiopia during the 2018/19 season. These data demonstrate that the prior ratification of a law that bans all alcohol advertisements on broadcast media by the Federal Government of Ethiopia in February 2019 with transitional period of 3 months and full implementation in May 2019 [13] is a timely public health intervention, and that alcohol advertising by foreign broadcasters using satellite broadcasts to Ethiopia may require monitoring. Overall, visual and verbal references to alcohol contributed 8.4% of the broadcast time included in the analysis.

This is, to our knowledge, the first study to measure alcohol advertising content to which televised EPL viewers are exposed in Ethiopia. This study was conducted after Ethiopia banned alcohol advertising in all local sports broadcasts and demonstrates that alcohol advertising persists in televised football, which is particularly popular among adolescents. The findings of this study would also serve as a baseline for future evaluation of the interventions. We included 19 matches from the EPL, broadcasted within a 1-month duration that would give adequate data for analysis of exposure to alcohol advertising. The double coding of two matches by another coder showed no inter-coder difference**.** We only considered games involving the five most popular teams of the EPL games as these would have the biggest audiences, but these considerations are also probably relevant to other televised national football leagues as well.

The proportion of programme time allocated to alcohol advertising was similar to a previous study analysing *Carlsberg* (a brand of beer) marketing in the Union of European Football Association (UEFA) Euro 2016 finals [18], and both the proportion and the duration of advertising in the active playtime were higher than observed in the UK in 2012 [3]. In this study, beer was the most commonly advertised type of alcohol in all matches accounting for 92.8% of the overall advertisement duration. This is consistent with previous studies that reported dominance of beer advertising in televised sports [3, 19]. However, the overall length of beer advertising in this study is longer than those previous studies.

The aim of any advertising is to increase awareness of products and brands, and ultimately to increase consumption, and systematic reviews of cohort studies conclude that this is effective [7, 20]. Our data from Ethiopian adolescents demonstrated that watching football on television was associated with drinking alcohol [16], and as football is very popular in Ethiopia, it represents an obvious opportunity to reach and influence this age group.

Unlike previous studies [3, 21], our analysis revealed that the great majority of alcohol advertising involved the addition of new images that were superimposed on the original broadcast game. These include adding a branded glass of beer or recognisable logo during the playtime or a group of young people smiling and looking relaxed together during out-of-play time. This may influence children and young adolescents watching the football together in ‘DSTV houses’ which are small local businesses with a satellite TV where people pay to watch international football games. A recent qualitative study from the capital of Ethiopia reported that the viewers of football games include children and young adolescents [4], who will thus inevitably be exposed to the repetitive alcohol adverts.

Ethiopia is a rapidly changing country with a relatively young population [22]. Alcohol has already been identified as a public health concern that may require population-based interventions to modify consumption [16, 23, 24]. Young people are particularly vulnerable as consuming alcohol may lead to higher levels of dependence and substance use in the future [25], depression [26] and suicidal behaviour [27].

Thus, regulating exposure to alcohol advertising via televised football games should be a public health priority for Ethiopia to promote lower levels of consumption and hence harm reduction in the future. A law that bans all alcohol advertisements on broadcast media was passed in February 2019 to be legally active 3 months from 29 May 2019 [13], yet these data, which were collected during the window between ratification and activation of this law, demonstrate that foreign satellite broadcasters will also require monitoring to ensure that they comply with the new Ethiopian national laws. Their acts further undermine the efforts undertaken by the government to reduce the short- and long- term harms associated with alcohol drinking. The images originate from the UK, where the Ofcom Broadcasting Code [28] restricts depictions of alcohol use in programmes made for children, and the glamorisation of alcohol use in programmes broadcast before the 9 p.m. watershed [29] otherwise likely to be widely seen, heard or accessed by children without editorial justification. However, the majority of alcohol advertising that is observed during EPL matches broadcast in Ethiopia are superimposed on the original images (Fig. 1a, b), and constitute a loophole in the current regulations, that may be available in other African countries as well as Ethiopia.

## Conclusions

There is a high baseline exposure to alcohol advertising during televised EPL games in Ethiopia just prior to the introduction of national laws prohibiting this in local mass media. As exposure to alcohol advertising increases future consumption [20], it is important to monitor if the national ban on alcohol advertising by overseas satellite broadcasters after the 29 May 2019 when the Ethiopian alcohol advertising ban is fully implemented [13]. As media broadcasters provide football games across Africa where they are very popular with young people, these observations are likely to be generalisable elsewhere in the continent.

## Data Availability

The original raw data used in this study are available from the corresponding author and can be presented upon reasonable request.

## Abbreviations

DSTV: Digital satellite television
EPL: English Premier League
TV: Television
UEFA: Union of European Football Association
WHO: World Health Organization
